# Improving adherence to acute low back pain guideline recommendations with chiropractors and physiotherapists: the ALIGN cluster randomised controlled trial

**DOI:** 10.1186/s13063-022-06053-x

**Published:** 2022-02-14

**Authors:** Simon D. French, Denise A. O’Connor, Sally E. Green, Matthew J. Page, Duncan S. Mortimer, Simon L. Turner, Bruce F. Walker, Jennifer L. Keating, Jeremy M. Grimshaw, Susan Michie, Jill J. Francis, Joanne E. McKenzie

**Affiliations:** 1 Department of Chiropractic, Faculty of Medicine, Health and Human Sciences, Macquarie University, Sydney, NSW 2109 Australia; 2 School of Public Health and Preventive Medicine, Monash University, 553 St Kilda Road, Melbourne, Victoria 3004 Australia; 3 Monash-Cabrini Department of Musculoskeletal Health and Clinical Epidemiology, Cabrini Health, Malvern, Victoria 3144 Australia; 4 Centre for Health Economics, Monash Business School, Monash University, Caulfield, 3145 Australia; 5 Discipline of Psychology, Exercise Science, Counselling and Chiropractic, Murdoch University, Murdoch, Perth, Western Australia 6150 Australia; 6 Department of Physiotherapy, Faculty Medicine Nursing and Health Sciences, Monash University, Clayton, Victoria 3800 Australia; 7 Clinical Epidemiology Program, Ottawa Hospital Research Institute; and Department of Medicine, University of Ottawa, Ottawa Hospital - General Campus, 501 Smyth Road, Ottawa, Ontario K1H 8L6 Canada; 8 Department of Clinical, Educational and Health Psychology, Centre for Behaviour Change, University College London, London, WC1E 7HB UK; 9 Division of Health Services Research & Management, School of Health Sciences, City University of London, Northampton Square, London, EC1V 0HB United Kingdom; 10 School of Health Sciences, University of Melbourne, Parkville, Victoria 3010 Australia

**Keywords:** Clinical practice guidelines, Implementation, Chiropractic, Physiotherapy, Low back pain

## Abstract

**Background:**

Acute low back pain is a common condition, has high burden, and there are evidence-to-practice gaps in the chiropractic and physiotherapy setting for imaging and giving advice to stay active. The aim of this cluster randomised trial was to estimate the effects of a theory- and evidence-based implementation intervention to increase chiropractors’ and physiotherapists’ adherence to a guideline for acute low back pain compared with the comparator (passive dissemination of the guideline). In particular, the primary aim of the intervention was to reduce inappropriate imaging referral and improve patient low back pain outcomes, and to determine whether this intervention was cost-effective.

**Methods:**

Physiotherapy and chiropractic practices in the state of Victoria, Australia, comprising at least one practising clinician who provided care to patients with acute low back pain, were invited to participate. Patients attending these practices were included if they had acute non-specific low back pain (duration less than 3 months), were 18 years of age or older, and were able to understand and read English. Practices were randomly assigned either to a tailored, multi-faceted intervention based on the guideline (interactive educational symposium plus academic detailing) or passive dissemination of the guideline (comparator). A statistician independent of the study team undertook stratified randomisation using computer-generated random numbers; four strata were defined by professional group and the rural or metropolitan location of the practice. Investigators not involved in intervention delivery were blinded to allocation. Primary outcomes were X-ray referral self-reported by clinicians using a checklist and patient low back pain-specific disability (at 3 months).

**Results:**

A total of 104 practices (43 chiropractors, 85 physiotherapists; 755 patients) were assigned to the intervention and 106 practices (45 chiropractors, 97 physiotherapists; 603 patients) to the comparator; 449 patients were available for the patient-level primary outcome. There was no important difference in the odds of patients being referred for X-ray (adjusted (Adj) OR: 1.40; 95% CI 0.51, 3.87; Adj risk difference (RD): 0.01; 95% CI − 0.02, 0.04) or patient low back pain-specific disability (Adj mean difference: 0.37; 95% CI − 0.48, 1.21, scale 0–24). The intervention did lead to improvement for some key secondary outcomes, including giving advice to stay active (Adj OR: 1.96; 95% CI 1.20, 3.22; Adj RD: 0.10; 95% CI 0.01, 0.19) and intending to adhere to the guideline recommendations (e.g. intention to refer for X-ray: Adj OR: 0.27; 95% CI 0.17, 0.44; intention to give advice to stay active: Adj OR: 2.37; 95% CI 1.51, 3.74).

**Conclusions:**

Intervention group clinicians were more likely to give advice to stay active and to intend to adhere to the guideline recommendations about X-ray referral. The intervention did not change the primary study outcomes, with no important differences in X-ray referral and patient disability between groups, implying that hypothesised reductions in health service utilisation and/or productivity gains are unlikely to offset the direct costs of the intervention. We report these results with the caveat that we enrolled less patients into the trial than our determined sample size. We cannot recommend this intervention as a cost-effective use of resources.

**Trial registration:**

Australian New Zealand Clinical Trials Registry ACTRN12609001022257. Retrospectively registered on 25 November 2009

**Supplementary Information:**

The online version contains supplementary material available at 10.1186/s13063-022-06053-x.

Contributions to the literature

- In implementation science, very few trials have been undertaken in the setting of physiotherapy and chiropractic.
- Despite a comprehensive theory-informed intervention of education and educational outreach that addressed barriers to the uptake of guideline recommendations, the intervention only led to changes in outcomes proximal to the intervention (i.e. predictors of clinician behaviour and intention) and an improvement for giving advice to stay active.
- The intervention did not change the primary study outcomes and was not cost effective.
- This study highlights the continuing challenges of implementing recommendations from evidence-based clinical practice guidelines into clinical practice.

## Background

Low back pain is a major individual and societal burden. Globally, low back pain is the number one contributor to years lived with disability and is in the top 10 contributors in every country in the world [1–3]. In Australia, the country where this study was undertaken, one in five people have low back pain at any given time, and four out of five Australians will experience low back pain at some point in their lives [4]. In 2011, the year this study was conducted, low back pain was the third leading cause of disease burden in Australia, accounting for approximately 4% of the total burden across all diseases and injuries [5], and in 2008–2009, AUD$1.2 billion was attributed to back problems, equal to 1.8% of total healthcare expenditure [5]. More recently, it was estimated that one in six Australians have chronic back problems and that these people report poorer quality of life than the general population [5].

Low back pain is one of the most common health problems seen in Australian primary care [6] and is the most common presentation to chiropractors [7]. Also, many people in Australia with low back pain attend physiotherapy clinics for treatment [8], with a recent survey showing that 61% of people with back pain attended a physiotherapist in the previous 12 months [9]. It is therefore essential that the care of people with low back pain presenting to Australian chiropractors and physiotherapists is based on the best available evidence. However, our research team has demonstrated that there is a need to improve professional practice in-line with guidelines in order to optimise the outcomes for people presenting with low back pain, including an overuse of X-rays by chiropractors in particular [10, 11].

In 2003, a multidisciplinary Australian clinical practice guideline for the diagnosis, prognosis and treatment of acute back pain was published and sent to all Australian primary care providers [12]. Key guideline recommendations were that (i) plain X-rays of the lumbar spine are not routinely recommended for people with acute non-specific low back pain as they are of limited diagnostic value and provide no benefits in pain, function or quality of life and (ii) advising patients to stay active produces a beneficial effect on pain, rate of recovery and function when compared to bed rest and a specific exercise regimen. Although this Australian guideline is now out of date, these recommendations remain current with more recent international guidelines for low back pain continuing to support these recommendations [13–15]. In addition to these recommendations, more recent guidelines have an increased focus on self-management, addressing the psychosocial issues of managing pain, and recommend stratified care approaches [16–18]. Successful implementation of guideline recommendations into clinical practice could improve outcomes for people with low back pain presenting to primary care providers (i.e. reduced pain and disability) and improve health system sustainability through reducing unnecessary imaging.

Implementation interventions evaluated to date for back pain have included different forms of clinician education, educational outreach visits, reminders, audit and feedback, or combinations of these delivered as multi-faceted interventions [19–23]. These systematic reviews found that audit and feedback, routine reminders, and multi-faceted interventions demonstrated some small positive effects on the quality of back pain management; however, the low number of high quality randomised trials means considerable uncertainty around these effects. Further, to the best of our knowledge, no implementation trials have been conducted in the chiropractic setting, and only one has been conducted in the physiotherapy setting [24], despite the common presentation of people with low back pain to these clinicians. Finally, previous implementation trials for low back pain have lacked a strong rationale for why particular interventions would be effective in certain settings [19].

The Theoretical Domains Framework (TDF) is a broad-based, comprehensive framework for designing implementation interventions offering coverage of potential change pathways [25, 26]. The original TDF comprises 12 domains and theoretical constructs synthesised from 33 theories and 128 theoretical constructs which can be used to identify theoretically and empirically informed explanations for implementation difficulties and to inform the design of implementation interventions [25, 27]. Few studies to date have used the TDF to explore barriers and facilitators and develop an implementation intervention tailored for chiropractors and physiotherapists who provide much of the management of low back pain in Australia [28, 29]. One suggested approach to design implementation interventions was developed by our team [30]. The approach comprised four steps framed as questions: step 1, who needs to do what, differently?; step 2, using a theoretical framework, which barriers and enablers need to be addressed?; step 3, which intervention components (behaviour change techniques and mode(s) of delivery) could overcome the modifiable barriers and enhance the enablers?; step 4, how can behaviour change be measured and understood? By answering these questions, implementation interventions with the aim of overcoming evidence-practice gaps can be developed utilising a systematic, theory-informed method.

## Aims

The primary objective of this cluster randomised trial was to estimate the effectiveness of a theory-based, systematically developed intervention that aimed to increase chiropractors’ and physiotherapists’ adherence to recommendations from a clinical practice guideline for acute low back pain, compared with passive dissemination of the guideline. Specifically, our primary objectives were to establish if the intervention was effective in:

(i) Reducing the percentage of patients with acute non-specific low back pain who were either referred for an X-ray, or received an X-ray, by increasing clinician adherence to the guideline recommendation about imaging (clinician behaviour);
(ii) Improving disability for patients three months post-onset of an episode of acute non-specific low back pain (patient level health outcome).

Secondary objectives included estimating the effects of the intervention for secondary outcomes in the following categories: (i) clinician behaviour (provided advice to stay active, advised bed rest, referred for imaging excluding X-ray); (ii) predictors of clinician behaviour (clinician intention to behave in a manner consistent with the guideline’s recommendations, behavioural constructs (e.g. knowledge, beliefs about capabilities)); (iii) patient health outcomes (pain severity, health-related quality of life); (iv) patient health behaviour (X-ray occurred); and (v) predictor of patient health behaviour (patients’ fear-avoidance beliefs (FAB)). We also determined whether this intervention was cost-effective.

## Methods

The protocol for the ALIGN (Acute Low back pain Implementing Guidelines iNto practice) trial has been published [31]. We provide an overview of the methods in this paper. Any deviations from the protocol are outlined in the Additional File, Table 1. The trial was retrospectively registered on the Australian New Zealand Clinical Trials Registry on the 25 November 2009 (ACTRN12609001022257); see the Additional File for the timeline of registration, and see Additional File, Table 2 for the labels used to describe outcomes across the trial report, published protocol and registry entry. The completed CONSORT checklist, and TIDieR reporting guideline checklist, are available as an Additional File.

### Trial design

This trial used a cluster randomised design with the clusters being chiropractic and physiotherapy practices with at least one practising clinician providing care to patients with acute low back pain.

### Recruitment of practices and clinicians

Recruitment of practices and clinicians occurred between November 2009 and February 2010. First, we placed notices about the trial in relevant professional physiotherapy and chiropractic newsletters to raise awareness of the study. Second, we approached all registered chiropractors and physiotherapists in Victoria, Australia, via mail (1196 chiropractors and 2463 physiotherapists). We sent each clinician an invitation letter and a maximum of four reminder letters, each 3 weeks apart. Third, we contacted a random selection of physiotherapists and chiropractors by telephone. Finally, when a clinician agreed to participate, a list of all clinicians employed at the same practice was created, and invitation letters were sent to the other clinicians informing them that the practice was included, encouraging them to participate, and allowing them to object to the practice participating if they wished. Incentives to participate included continuing professional development points, payment (AUD$5 per patient) for assistance in accessing clinical files of included patients, and entry into a draw to win a prize to attend a professionally-relevant Australian conference (up to a maximum AUD$800).

### Recruitment of patients

Clinicians determined if the patient met the trial’s inclusion criteria (see below). Patients then consented to data collection, but not the intervention, because the intervention was directed at the clinician level. Consenting patients with acute non-specific low back pain who met inclusion/exclusion criteria were considered patient participants.

Over a 2-week period, at least 3-months after intervention delivery (between May and September 2010), clinicians approached all consecutive patients for consent to participate. We limited this time period to 2 weeks because we anticipated that clinicians would not tolerate the extra burden of having to approach consecutive patients for any longer than this. Patients did not need to be seeing clinicians for the first time during the 2-week period; they just needed to meet the inclusion criteria.

At the end of the consultation, the clinician provided eligible patient participants with additional documentation regarding ongoing participation in the trial. This participation comprised additional data collection, either through completion of a survey at 3-months post onset of acute low back pain, or allowing their clinical file to be audited by the research team, or both. To promote participation in additional data collection, patients were offered the opportunity to enter a draw to win a mobile/cell phone.

### Inclusion and exclusion criteria

Chiropractic and physiotherapy practices were included if at least one clinician within the practice provided written informed consent, the practice was located in the state of Victoria, Australia, and there were no objections to participation from other clinicians in the practice.

Patient participant inclusion criteria, determined by the clinician, were as follows: the patient attended a participating clinician for acute non-specific low back pain, with pain duration less than 3 months; provided consent; were 18 years of age or older; and were able to understand and read English. Patients attending the enrolled practices were not eligible if any of the following criteria were met: radicular leg pain was present (neurological signs and symptoms); previous spinal surgery; ‘red flags’ were present alerting the possibility of serious pathological conditions such as malignancy, infection, or fracture; or, they were pregnant.

### Randomisation and allocation concealment

A statistician independent of the trial team undertook the randomisation after we provided him with only practice identification codes and stratification variables (i.e. no practice identifying information was provided). Four strata were defined by professional group (chiropractors or physiotherapists) and whether the practice was in a rural or metropolitan location, defined from the Rural, Remote and Metropolitan Areas classification system [32]. Within each stratum, practices were allocated to the intervention and comparator groups with equal probability (1:1 randomisation ratio). Practices were randomly allocated at a single point in time by generating a computer random number for each practice, sorting on the random number within each stratum, and allocating every alternate practice to the intervention group. Allocation was concealed from the investigators until baseline data had been collected from clinicians.

### Blinding

Due to the nature of the intervention, it was not possible to blind the clinicians to group allocation; however, clinicians only received minimal information about the intervention content in the recruitment material. Patient participants were informed that their clinician was participating in a study assessing clinicians’ management of patients presenting with acute back pain, but they were not informed of the study design, nor of their clinicians’ group allocation. The following individuals were blinded to group allocation: outcome assessors who extracted data from clinical files of patients, research assistants entering data from clinician and patient completed checklists and questionnaires, and the statistician who undertook the data analysis (SLT). The following individuals were not blinded to group allocation: investigators involved in the delivery of the intervention and the statistician who designed the trial (JEM).

### Interventions

In addition to the guideline’s existing dissemination strategy, comparator group clinicians received a printed copy of the summary of the guideline and a written reminder of how to access the guideline electronic version. Comparator group materials were sent to clinicians in March 2010.

A detailed description of the content of the ALIGN tailored, multi-faceted intervention is included in the Additional File, Table 3. To develop the intervention, we used the TDF to identify barriers and facilitators to the target behaviours and guide the design of the ALIGN implementation intervention [25, 30]. First, barriers to, and facilitators of, clinician behaviour change in line with the guideline recommendations were identified via semi-structured interviews, and a subsequent survey, of practising physiotherapists and chiropractors. The identified barriers and facilitators were thematically mapped to the TDF to enable identification of six hypothesised determinants of change (beliefs about capabilities, beliefs about consequences, social influences, professional role, knowledge, and intentions) [27]. Next, we selected eight behaviour change techniques (BCTs) from the Theory-Technique Matrix considered most effective in changing the hypothesised determinants [33], including the following: increasing skills; rehearsal of relevant skills; social processes of encouragement, pressure and support; feedback; persuasive communication; information regarding behaviour, outcome; modelling; and information provision. These BCTs were incorporated into four intervention components as follows:

1. A full-day interactive symposium-style event comprising didactic lectures relevant to the guideline recommendations delivered by peer opinion leaders, small group discussions led by trained clinical facilitators, and practical sessions and rehearsal with simulated patients. In each component of the symposium, different BCTs were delivered. Separate symposia were held for physiotherapists and chiropractors and took place on 20 and 27 February 2010, respectively. More detailed information about the symposia are available in the ALIGN protocol [31].
2. All clinicians in the intervention group, including those who were not able to attend the symposium, received a DVD that included videos of the symposium didactic sessions;
3. Supporting written educational material;
4. Academic detailing, comprising a scheduled follow-up telephone call with a clinical member of the project team to discuss difficulties encountered in implementing behaviour change, and strategies to overcome these difficulties.

The BCTs delivered in the intervention are detailed in the Additional File, Table 3 (ALIGN intervention components). These BCTs were conceptualised as the ‘active ingredients’ of the intervention. BCTs were selected from the Theory-Technique Matrix (TTM; a precursor of the BCT taxonomy (BCTTv1) [34]) to target the hypothesised determinants of change generated from interviews and a survey of practising physiotherapists and chiropractors. Two behavioural health psychologists (SM and JF) and an implementation researcher (DOC) reviewed the results from the interviews and survey and considered the target behaviours and theoretical domains we were trying to change (domains predictive of intention), and the BCTs considered applicable/relevant to alter or redirect the target domains, as advocated by the TTM. These BCTs were then discussed and developed into an intervention over several local investigator meetings, comprising physiotherapy and chiropractic clinical champions and implementation scientists. The ALIGN investigator team iteratively developed the intervention matrix (this documented how the BCTs were operationalised in the intervention) and accompanying intervention materials, both of which were presented to our advisory committee that comprised implementation scientists and clinicians, and was refined in response to this. We also sought feedback on the intervention matrix and materials from international colleagues with expertise in this area and refined the intervention in response to this; we asked about relevance to physiotherapists and chiropractors, acceptability, if this was likely to change clinician behaviour, and anything we may have missed.

### Study outcomes

The study outcomes, data collection methods, and assessment periods are listed in Table 1.

**Table 1:**
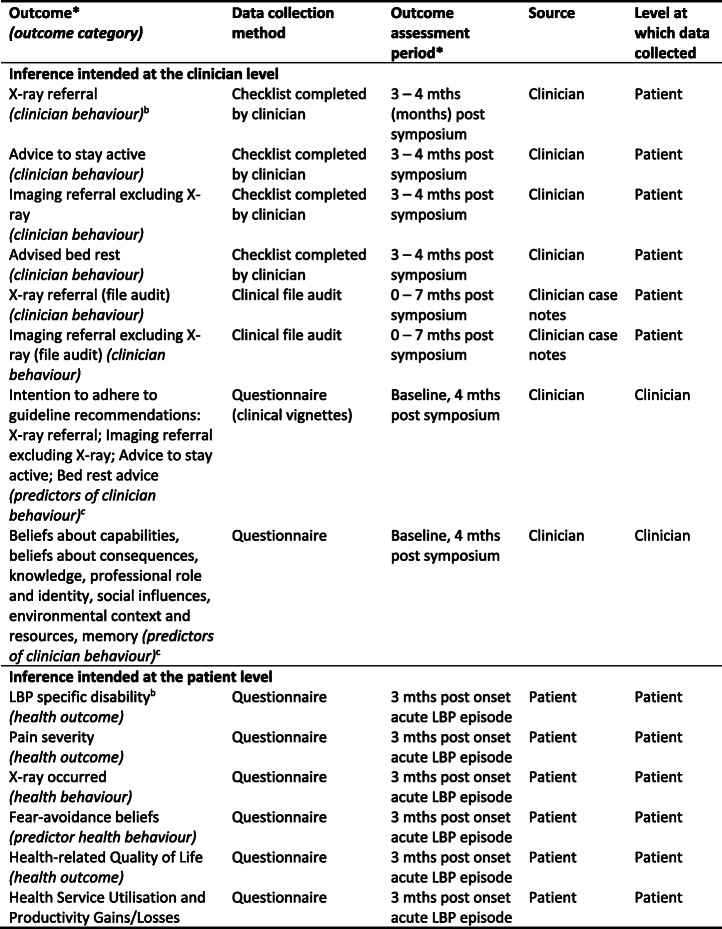
Outcome measures^a^ reported in trial

*All outcomes and time points consistent with trial registry entry. See Additional File, Table 2 for detail about labels used to describe outcomes across the trial report, protocol and registry entry

^a^Table adapted from protocol publication [32]

^b^Primary outcome

^c^For a full description of these secondary outcomes, see published protocol table “Details of the outcome measurement for the behavioural constructs” (additional file #3)

#### Data collection

Data were collected using a range of methods. Baseline clinician data, including demographic information, intention via clinical vignettes and behavioural constructs, were collected at the time of enrolment. Clinicians completed a patient encounter form to record diagnostic imaging procedures undertaken or ordered in that encounter. At the end of their consultation, patient participants completed a checklist to record interventions and diagnostic procedures they received. Multiple encounter forms could have been completed for the same patient if they visited the clinician on multiple occasions during the recording period. Therefore, basic patient demographic information was recorded on patient encounter forms and cross-checked so this could be accounted for in the analysis. File audits also documented evidence of the patient being referred for lumbar spine imaging at any time during their care for this episode of low back pain and the type and date/s of referral/conduct of imaging. The definitions of how multiple encounters were handled are outlined in the published protocol.

Both clinician and patient participants also completed questionnaires; clinicians completed questions about the predictors of clinician behaviour and patients about pain and disability. For each patient, we measured the start date of their episode of low back pain in the baseline questionnaire and then provided the follow-up questionnaire 3 months after this date.

Finally, at urban practices, we undertook clinical file audit of consenting participating patients’ files for the time period commencing from when their practitioner attended the intervention symposium, up to 7 months post-intervention. This file audit extracted information about whether imaging was taken by the practitioner, or if the patient was referred for imaging, of either lumbosacral plain X-ray, full spine plain X-ray, or other types of imaging, e.g. CT scan, MRI, bone scan. Evidence of referral included any of the following: referral letter to a general practitioner, referral noted, imaging report, findings noted, or other evidence of either referral or imaging.

#### Primary outcomes

The primary outcome at the clinician level measured the effectiveness of the intervention in changing the clinicians’ behaviour for the guideline recommendation about X-ray referral. This was measured over a 2-week data collection period via a clinician-completed checklist, to determine whether the clinician ordered, undertook, or recommended a lumbar X-ray for patients with acute non-specific low back pain. The primary outcome at the patient level was low back pain-specific disability 3-months post-onset of their acute low back pain episode, measured with the Roland-Morris Disability Questionnaire (RDQ) by telephone survey [35, 36]. We considered it was important to measure patient level outcomes in this trial because it was unclear whether the implementation intervention would result in a change in the patient’s health status. Trials which underpin the key message from the guideline on providing advice to stay active differ in regard to the interventions employed, delivery of the intervention and control arms, and have only shown small beneficial effects for the outcomes pain, rate of recovery and function.

#### Secondary outcomes

Secondary outcomes included the following:

1. *Measures of clinician behaviour:* provided advice to stay active; advised bed rest; referred for imaging excluding X-ray
2. *Predictors of clinician behaviour: intention* to behave in a manner consistent with the guideline recommendations, *knowledge* of the guideline and how to perform the guideline-recommended behaviours, *beliefs about capabilities* (feeling confident they can perform the guideline-recommended behaviours), *beliefs about consequences* (believing that performing in this way will lead to positive outcomes), *professional role and identity* (i.e. believing it is their professional responsibility to behave in this way), *social influences* (i.e. feeling social pressure to behave in this way), *environmental context and resources* (i.e. perceiving their environment supports behaviour consistent with the guidelines), *memory* (i.e. remembering to behave in this way), and *fear-avoidance beliefs* about physical activity
3. *A patient health behaviour:* attended a radiology clinic and received an X-ray
4. *A predictor of patient health behaviour:* fear-avoidance beliefs
5. *Patient health outcomes:* pain severity; health-related quality of life.

#### Intervention fidelity

Coverage, frequency, and duration of the intervention were measured by documenting what proportion of the practitioners in the intervention group attends the workshops, the frequency of the workshops, and the duration of each workshop. At each symposium, an independent assessor completed a fidelity checklist to determine if the intervention elements, and BCTs, were delivered as planned. We also measured how many practitioners reported viewing the DVD of the symposium, and what percentage of practitioners received the follow-up phone call (educational outreach).

### Sample size

Full details of the sample size calculation are provided in the protocol [31]. Briefly, to provide 80% power to detect a difference of 10% in X-ray referral between intervention groups, we estimated that we would require 136 practices (68 physiotherapy and 68 chiropractic practices), with each completing checklists for an average of 20 patient participants, providing a total of 2720 patient participants. This assumed a 39% X-ray referral rate in the comparator group, a 5% significance level, an intra-cluster correlation coefficient (ICC) of 0.10, and allowed for 20% attrition in practices. We chose the ICC of 0.10 based on previous research that suggested ICCs of the order of 0.10 for process variables, such as X-ray referral, in primary care [37]. X-ray referral rates were not available for the Australian context when we determined the sample size, so the X-ray referral rate of 39% was estimated from international research. For chiropractors, several studies estimated that referral for X-ray for acute low back pain ranged from 62 to 72% [38–40]. For physiotherapists, who often treat patients with back pain on referral from, and in conjunction with, general medical practitioners, we assumed that X-ray referral rates would be similar to those found in Australian general practice, which was estimated at 28% [41]. Because we intended to recruit an equal number of patient participants from physiotherapy and chiropractic practices, we estimated the pooled X-ray referral rate to be 47%. Interventions comparing standard guidelines dissemination with no intervention control groups have shown improvements in care of approximately 8% [42]; hence, we anticipated a decrease in the percentage of X-ray referral in the control group of this magnitude, providing an estimated referral rate of 39%.

### Analyses

#### Primary analyses

In line with our published analysis plan [31], we undertook a modified intention-to-treat (ITT) analysis as our primary effectiveness analysis, where we analysed clusters and participants (clinicians and patients) as they had been randomised, but did not impute missing data. We estimated the effectiveness of the intervention on primary and secondary outcomes with marginal modelling *of individual patient data* using generalised estimating equations (GEEs).

Descriptive statistics of demographic and potential confounding variables are presented at baseline. We estimated the effectiveness of the intervention on primary and secondary outcomes with marginal modelling of individual patient data using GEEs. We fitted an exchangeable correlation structure, where responses from the same practice were assumed to be equally correlated [43]. Additionally, we used robust variance estimation to provide valid standard errors even if the within-cluster correlation structure was incorrectly specified [44]. For binary outcomes, a logit link function was used. In the event where the ICC of an outcome for a particular analysis was negative, we refitted the GEE with an independent correlation structure, which assumes an ICC of zero. Our primary analyses of outcomes adjusted for stratification variables (professional group and location of the practice) and pre-specified potential confounding variables. All pre-specified confounders were included in the models even when no baseline imbalance existed. In circumstances where there were limited data or events, or both, to adjust for all confounders, we report estimates of intervention effect from unadjusted models or models adjusted for only the stratification variables. Details of changes to the variables adjusted for are available in Additional File, Table 1.

Estimates of intervention effect from these models with binary outcomes yield odds ratios. In addition to the odds ratios, we also provide estimates of risk difference to aid interpretability and provide greater information to fully assess the implications of the intervention effects [45]. Marginal standardisation was used to estimate the risk differences. This involved using the estimated regression coefficients from the fitted GEEs to calculate average predicted proportions with the outcome in each intervention group, from which, risk differences were calculated [46]. For each outcome, the estimate of intervention effect and its 95% CI are provided. For the primary outcomes (X-ray referral, low back pain-specific disability), we provide estimates of ICCs and their 95% CIs. ICCs were calculated using the analysis of variance method. For the dichotomous primary outcome (X-ray referral), the confidence interval was bootstrapped using the combination of the bootstrap and the loneway commands in Stata. We allowed for clustering of observations within practices. A bias corrected 95% confidence interval was calculated using 1000 replicates. Regression diagnostics were used to assess the influence of outliers on estimates of intervention effect and for analysing residuals. No adjustment was made for multiple testing. All tests were two-sided and carried out at the 5% level of significance. Analysis additions and deviations from the protocol are outlined in Additional File, Table 1.

#### Secondary analyses

We conducted a subgroup analysis to examine whether the effect of the intervention on X-ray referral was modified by professional group (physiotherapist or chiropractor). We examined this by fitting a model that included an interaction term between intervention group and professional group. The estimated ratio of odds ratios and its 95% CI are presented. As part of the secondary analyses, for the predictors of clinician behaviour outcomes, we examined whether clinician and practice characteristics (age, professional group, Gonstead practitioner, self-reported special interest in low back pain, number of practitioners per practice, location of the practice, and baseline measure of the outcome) were potential predictors of missing data through modelling. All variables were included in the primary analysis model. We undertook a sensitivity analysis to investigate the impact of allowing for clustering at the level of the clinician, which showed no important impact (results not shown).

#### Economic evaluation

Planned analyses included an economic evaluation alongside the ALIGN trial [31]. This economic evaluation was designed to quantify the additional costs (savings) arising from the ALIGN intervention as compared to access to the guideline via existing practice. Estimates of additional costs (savings) were then to be compared against estimated treatment effects with respect to clinical practice and patient health outcomes to evaluate the cost-effectiveness of the ALIGN intervention as compared to existing practice. ALIGN would represent a cost-effective use of resources if:

(i) The direct cost of the ALIGN intervention were fully offset by reductions in health service utilisation and/or productivity gains (i.e. ALIGN is cost-saving) *and* ALIGN was no worse than existing practice on clinical practice and patient health outcomes; or
(ii) ALIGN was not cost-saving *but* the additional (net) costs of ALIGN were outweighed by additional benefits (i.e. treatment effects with respect to clinical practice and patient health outcomes).

Cost-savings required to establish (i) were hypothesised to flow directly from implementation of guideline recommendations to limit referral for imaging (fewer x rays, CT scans and MRIs). However, productivity gains and reductions in use of primary care, allied health, and pharmaceuticals were also possible if adherence to guideline recommendations accelerated recovery and improved patient health outcomes. If results failed to demonstrate a treatment effect in favour of the intervention group with respect to X-ray referral (primary outcome), X-ray occurred, or other imaging referral (secondary outcomes), then the main mechanism of action for a reduction in health service utilisation could be excluded. If results also failed to demonstrate a treatment effect with respect to low back pain-specific disability (primary patient health outcome) and pain severity (secondary patient health outcome), then we would be left with no plausible explanation for cost-offsets from reduced health service utilisation and productivity gains. Put simply, results from the main effectiveness analysis may allow us to exclude the possibility that ALIGN is cost-saving *without proceeding to a full economic evaluation.*

Improvements in clinical practice or patient health outcomes required to establish (ii) should also be evident from the main effectiveness analysis. If ALIGN is not cost-saving *and* the main effectiveness analysis fails to demonstrate a treatment effect on primary (X-ray referral) and secondary (any imaging referral, low back pain-specific disability and quality-adjusted life-years) outcomes for the economic evaluation, then calculating incremental cost-effectiveness ratios with respect to these outcomes (as per our analysis plan) *cannot demonstrate cost-effectiveness*.

Findings from the main effectiveness analysis were sufficient to draw conclusions regarding cost-effectiveness. We therefore present a summary of costs and consequences rather than a full accounting of incremental cost-effectiveness. This deviation from the protocol simplified our analyses and simplified interpretation of results.

## Results

We received a better than expected response to recruitment of practices with 210 practices agreeing to participate, comprising 133 physiotherapy practices (180 physiotherapists), and 77 chiropractic practices (88 chiropractors). Given the uncertainty in the parameters informing our sample size calculation (e.g. ICC), we included all interested practices.

**Fig. 1:**
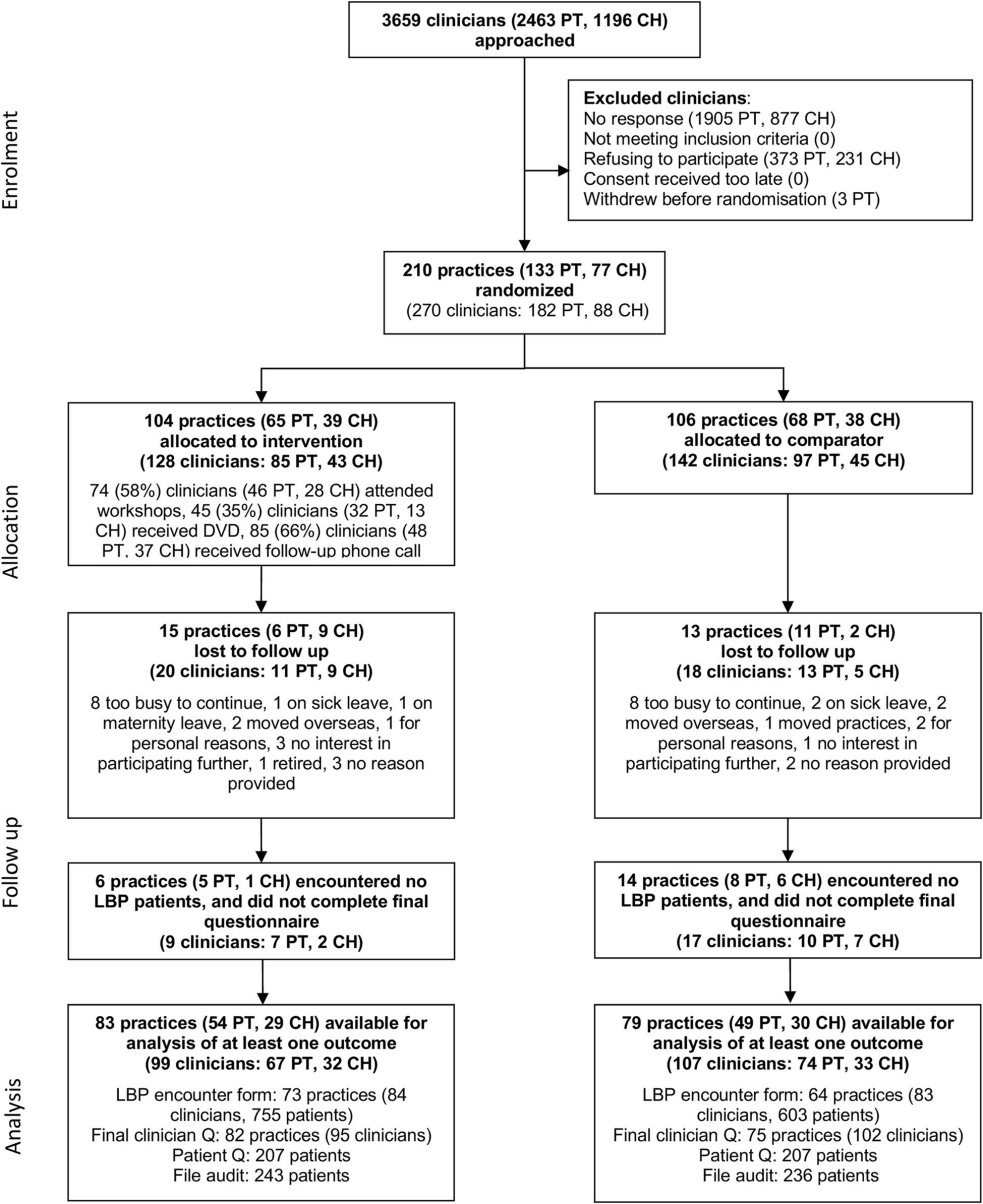
Flow of practices and patient participants through the ALIGN cluster randomised trial

Flow of practices, clinicians, and patients through the trial is shown in Fig. 1. Overall 162 practices (77%) and 206 clinicians (76%) were available for the analysis of at least one outcome. For the patient-level primary outcome, 449 patients from 106 practices were included in the analysis; this is much smaller than the anticipated 20 patients per practice (n = 2,720) in our original sample size calculation [31].

Practice and clinician baseline characteristics are shown in Table 2 and Table 3. There was some baseline imbalance in practice characteristics with intervention group practices more likely to have an X-ray facility on site (15% versus 9%) and more likely to have access to a bulk-billing, government funded, and radiology service (83% versus 74%) (Table 2). Baseline clinician intention to adhere to the guideline recommendations indicated that the intervention group clinicians were more likely to not adhere to the X-ray referral recommendation (42% versus 35%) (Table 3). Other baseline hypothesised predictors of baseline clinician behaviour were similar between the groups (Additional File, Table 4). Data from the clinician checklist indicated that included patients were similar between groups (Table 4). For those patients who responded to the follow-up survey, there was some baseline difference with patients in the intervention group more likely to be compensable (10% versus 3%) (Table 5).

**Table 2:**
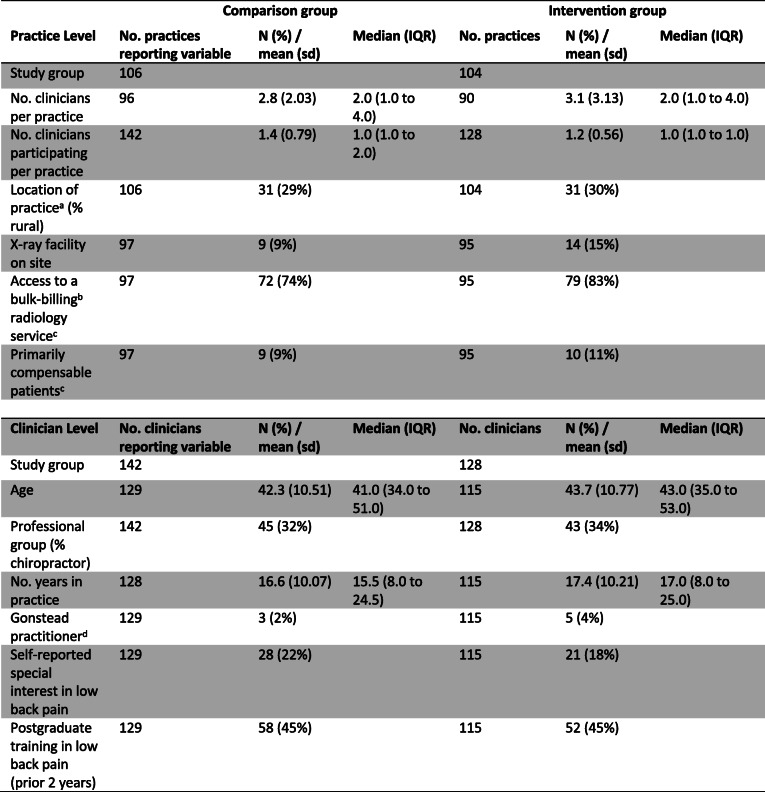
Baseline values for practice level and clinician level data

*sd* standard deviation, *IQR* interquartile range

^a^Location of practice (urban/rural) and Professional group (physiotherapist/chiropractor) were the stratification variables

^b^Bulk billing: the total payment for patient’s consultation is paid for by the Medicare system

^c^These variables were allowed to vary within practice (10 differences in bulk billing, 6 in compensable patients). Primarily compensable patients refers to whether clinicians answered yes to the question “Do you primarily treat Work Cover (compensable) patients at your practice”

^d^As a subset of the chiropractic profession, the comparison group had 3/38 (7%) and the intervention group had 5/34 (13%) Gonstead practitioners (a type of chiropractic practice where clinicians typically use routine X-rays)

**Table 3:**
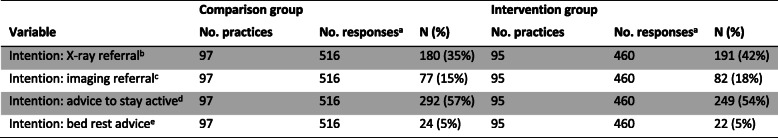
Baseline clinician intention to adhere to guideline recommendations as measured by clinician-completed vignettes

^a^Each clinician responded to four vignettes

^b^Was coded ‘Yes’ if the clinician ticked either “Lumbosacral plain X-ray” or “Full spine pain X-ray” in the vignette response questionnaire

^c^Was coded ‘Yes’ if the clinician ticked “Lumbar CT scan”, “Lumbar MRI”, or “Bone Scan” in the vignette response questionnaire

^d^Was coded ‘Yes’ if the clinician ticked “Advice to stay active” in the vignette response questionnaire

^e^Was coded ‘Yes’ if the clinician indicated “Bed rest” for greater than two days in the vignette response questionnaire

**Table 4:**
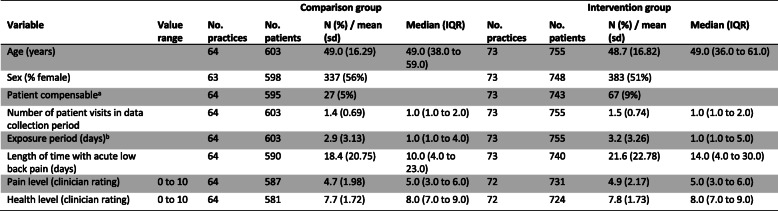
Summary data of all patients (collected from the clinician checklist)

*sd* standard deviation, *IQR* interquartile range.

^a^Primarily compensable patients referred to whether the costs associated with the injury were covered by workers’ compensation

^b^Exposure period refers to the number of days between first and last visit (inclusive)

**Table 5:**
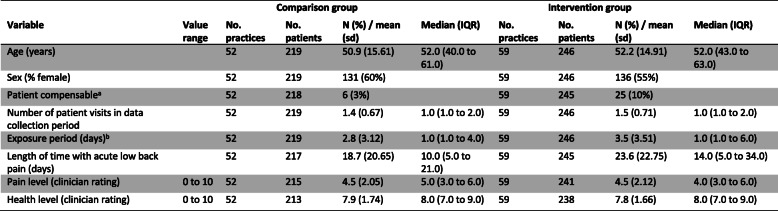
Summary data of the patients who responded to the 3-month follow-up survey

*sd* standard deviation; *IQR* interquartile range

^a^Primarily compensable patients refers to whether the costs associated with the injury are covered by workers’ compensation

^b^Exposure period refers to the number of days between first and last visit (inclusive)

**Fig. 2:**
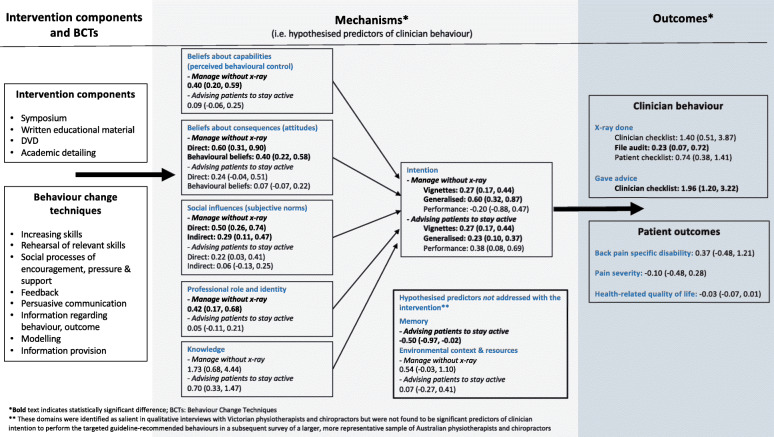
ALIGN logic model

The ALIGN Logic Model is shown in Fig. 2. This model outlines the selected BCTs hypothesised to modify or redirect the barriers to behaviour change. The model shows the results along the continuum of behaviour change from hypothesised predictors of clinician behaviour and patient outcomes.

In terms of fidelity of intervention delivery, 74 clinicians (58%; 46 physiotherapists and 28 chiropractors) randomised to the intervention group attended the intervention symposium. All clinicians in the intervention group were provided with the supporting written educational material and the DVD. Forty-five clinicians (35%; 32 physiotherapists and 13 chiropractors) self-reported viewing the DVD, and 85 clinicians (66%; 48 physiotherapists and 37 chiropractors) received the follow-up telephone call. As documented by the independent assessor completing a fidelity checklist, 43% (3/7) of intervention elements and 57% (21/37) of BCTs were delivered as planned at the physiotherapy symposium, and 86% (6/7) of intervention elements and 76% (28/37) of BCTs were delivered as planned at the chiropractic symposium. Intervention elements not delivered as planned in the physiotherapy symposium were as follows: skills demonstration session on effectively communicating with patients and giving advice to stay active, small group practical to rehearse diagnostic and communication skills with simulated patients, repeat straw poll, and reflective activity. In the chiropractic symposium, the small group practical to rehearse diagnostic and communication skills with simulated patients was not delivered as planned.

### Effectiveness of the intervention

#### Primary outcomes

There was no important difference between groups in the odds of patients being referred for X-ray as measured by the clinician-completed checklist (adjusted (Adj) OR: 1.40; 95% CI 0.51, 3.87; Adj risk difference (RD): 0.01; 95% CI − 0.02, 0.04) (Table 6). There was no important clinical difference in low back pain-specific disability between groups (Adj mean difference: 0.37; 95% CI − 0.48, 1.21; scale 0 to 24) (Table 7). The estimated ICC for referral for X-ray was 0.15 (95% CI 0.09 to 0.17) and for low back pain-specific disability was 0 (95% CI 0 to 0.07).

**Table 6:**
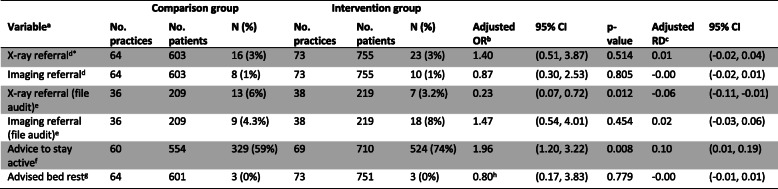
Estimated effects of the intervention on clinician behaviour outcomes (clinical checklist and clinical file audit)

*Primary outcome

^a^X-ray referral was coded ‘Yes’ if the clinician ticked either “Lumbosacral plain X-ray” or “Full spine pain X-ray” at any of the patient consultations over the two week data collection period. Imaging referral was coded ‘Yes’ if the clinician ticked “Lumbar CT scan”, “Lumbar MRI”, or “Bone Scan” at any of the patient consultations over the two week data collection period. Advice to stay active is coded ‘Yes’ if the clinician ticks “Advice to stay active” at any of the patient consultations over the two week data collection period. Advised bed rest is coded ‘Yes’ if the clinician indicated “Bed rest” for greater than 2 days at any of the patient consultations over the 2 week data collection period

^b^Adjusted effects from models fitted using generalised estimating equations analysis with exchangeable correlation (unless otherwise noted) structure and robust variance estimation to allow for clustering within practices. OR = odds ratio

^c^*RD* risk difference. RD calculated from marginal probabilities. Confidence intervals were calculated using a pairwise comparison of margins after fitting a GEE model using Stata, allowing for clustering of observations within practices.

^d^X-ray referral and imaging referral outcomes were only adjusted for the stratification variables, professional group (physiotherapist/chiropractor) and location of practice (urban/rural) due to high rates of adherence (resulting in low event rates)

^e^Adjusted for: stratification variables (professional group, location of practice), patient level (age, sex, LBP compensation), clinician level (age, Gonstead practitioner, years in practice, special interest in LBP, postgraduate training, baseline intention (X-ray or imaging referral, as appropriate), and practice level (bulk billing, X-ray on site, compensable patients, number of clinicians in the practice). The pre-specified confounding variables ‘number visits for this episode of acute LBP’ and ‘≥ 1 x-ray LBP previous 12 mths’ were not adjusted for (see Additional file 1)

^f^Adjusted for: stratification variables (professional group, location of practice), patient level (age, low back pain compensation, number of patient visits in the data collection period, exposure period, length of time with acute low back pain), clinician level (age, Gonstead practitioner, years in practice, special interest in low back pain, postgraduate training, baseline intention advice to stay active), and practice level (number of clinicians in the practice) (Fig. 2 of the trial protocol [22])

^g^Advised bed rest outcome is unadjusted for the stratification variables and pre-specified confounders due to a limited number of events

^h^Modelled with an independent correlation structure

**Table 7:**
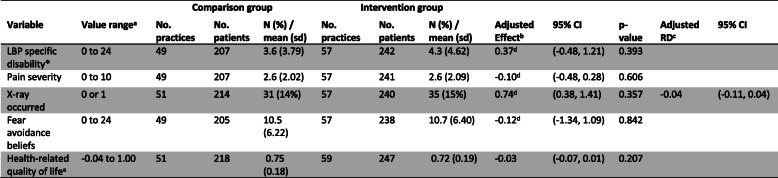
Estimated effects of the intervention on patient outcomes (3 months)

*LBP* low back pain, *sd* standard deviation, *IQR* interquartile range

*Primary outcome

^a^The value ranges for LBP specific disability are based on the Roland-Morris Disability Questionnaire; higher values indicate higher levels of disability. Pain severity is measured using a modified version of the characteristic pain intensity subscale of the Graded Chronic Pain Scale, higher values indicate higher levels of pain. Fear avoidance beliefs will be measured using the Fear Avoidance Beliefs Questionnaire physical activity subscale, higher values reflect greater fear avoidance. AQoL-4D utility scores are anchored at death (0.00) and full health (1.00) and scaled from − 0.04 to 1.00 where negative utility values designate states worse than death

^b^Adjusted effects from models fitted using Generalised Estimating Equations with exchangeable correlation structure and robust variance estimation to allow for clustering within practices. Effect estimates are the difference in means, with the exception of the outcome ‘X-ray occurred’, where the effect estimate is an odds ratio. Models adjusted for pre-specified confounding variables noted in Fig. 2 of the trial protocol [22], except for the confounding variables ‘number visits for this episode of acute low back pain’ and ‘≥ 1 X-ray low back pain previous 12 mths’ (see Additional file 1)

^c^*RD* risk difference. RD calculated from marginal probabilities. Confidence intervals were calculated using a pairwise comparison of margins after fitting a GEE model using Stata, allowing for clustering of observations within practices

^d^Modelled with an independent correlation structure

^e^AQoL-4D utility scores

#### Subgroup analysis

In the subgroup analysis for X-ray referral, the effects of the intervention were different by professional group (chiropractors OR: 1.85; 95% CI 0.58, 5.91; physiotherapists: OR: 0.34; 95% CI 0.04, 2.93); however, the confidence interval for the ratio of odds ratios was wide providing no clear evidence of a difference between professional groups (ratio of OR: 5.52; 95% CI 0.47, 64.5; *p*-value = 0.17).

#### Secondary outcomes

Table 6 shows the results for the clinician-completed checklist and the file audit (measures of clinician behaviour). There was no clear evidence of a difference between groups for X-ray referral or imaging referral for the checklist-measured outcomes. For the file audit data, intervention group clinicians were less likely to refer for X-ray (Adj OR: 0.23; 95% 0.07, 0.72; Adj RD: − 0.06; 95% CI − 0.11, − 0.01), but there was no clear evidence of a difference for overall imaging referral (Adj OR: 1.47; 95% CI 0.54, 4.01; Adj RD: 0.02; 95% CI − 0.03, 0.06). Patients in the intervention group were more likely to be given advice to stay active (Adj OR: 1.96; 95% CI 1.20, 3.22; Adj RD: 0.10; 95% CI 0.01, 0.19). There was no difference between groups for bed rest advice.

Table 7 shows the results for the patient level outcomes. There were no important differences between groups in any of the secondary patient level outcomes, including pain severity, fear-avoidance beliefs, and whether the patient reported that an X-ray had occurred at 3 months follow-up.

When responding to patient vignettes on the post-intervention questionnaire (Table 8), intervention group clinicians were more likely to intend to adhere to the guideline for X-ray referral, with lower odds for X-ray referral intention (Adj OR: 0.27; 95% CI 0.17, 0.44; Adj RD: − 0.16; 95% CI − 0.22, − 0.11) and more likely to give advice to stay active (Adj OR: 2.37; 95% CI 1.51, 3.74; Adj RD: 0.16; 95% CI 0.09, 0.24). There was no clear evidence of a difference in intention related to general imaging referral (Adj OR 0.61; 95% CI 0.32, 1.15) or bed rest advice (Adj OR 2.22; 95% CI 0.68, 7.26).

**Table 8:**
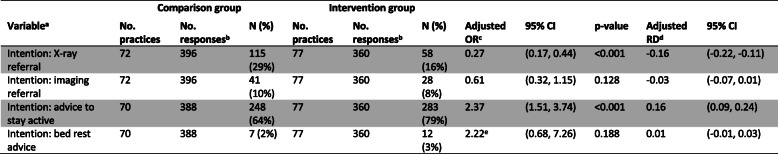
Estimated effects of the outcome on clinicians’ intention to adhere to guideline recommendations (clinician post-intervention questionnaire – vignettes)

^a^Intention: X-ray referral is coded ‘Yes’ if the clinician ticked either “Lumbosacral plain X-ray” or “Full spine pain X-ray” in the vignette response questionnaire. Intention: imaging referral is coded ‘Yes’ if the clinician ticked “Lumbar CT scan”, “Lumbar MRI”, or “Bone Scan” in the vignette response questionnaire. Intention: advice to stay active is coded ‘Yes’ if the clinician ticked “Advice to stay active” in the vignette response questionnaire. Intention: advised bed rest is coded ‘Yes’ if the clinician indicated “Bed rest” for greater than 2 days in the vignette response questionnaire

^b^Each clinician responded to four vignettes

^c^Adjusted effects from models fitted using Generalised Estimating Equations with exchangeable correlation structure (unless otherwise noted) and robust variance estimation to allow for clustering within practices. OR = odds ratio. Models adjusted for pre-specified confounding variables noted in Fig. 2 of the trial protocol [22]

^d^*RD* risk difference. RD calculated from marginal probabilities. Confidence intervals were calculated using a pairwise comparison of margins after fitting a GEE model using Stata, allowing for clustering of observations within practices

^e^Modelled with an independent correlation structure

Table 9 shows the results for other hypothesised predictors of clinician behaviour. For the clinical behaviour ‘managing patients without an X-ray’, there was a statistically significant difference between groups in *Intention (generalised)*, *Beliefs about capabilities*, *Beliefs about consequences (direct and behavioural beliefs), Professional role and identity*, and *Social influences (direct and indirect)*. This demonstrates clinicians in the intervention group had greater intention to manage patients without X-ray, felt more confident doing so, had stronger beliefs that this would lead to positive outcomes, had stronger beliefs that this was their professional responsibility, and felt greater social pressure to manage patients without X-ray compared with clinicians in the comparator group.

**Table 9:**
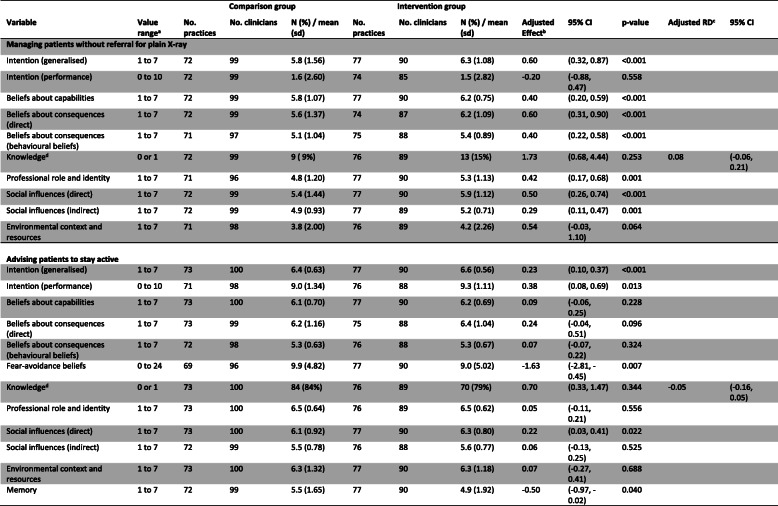
Estimated effects of the outcome on hypothesised predictors of clinician behaviour (clinician post-intervention questionnaire –behavioural constructs)

*sd* standard deviation

^a^For all outcomes (except fear avoidance beliefs), a larger score indicates greater agreement or likelihood in the clinicians’ intentions and beliefs in performing the particular behaviour (i.e. not referring for plain X-ray or advising patients to stay active)

^b^Adjusted effects from models fitted using generalised estimating equations with exchangeable correlation structure and robust variance estimation to allow for clustering within practices. Effect estimates are the difference in means, with the exception of the outcome ‘knowledge’, where the effect estimate is an odds ratio. Models adjusted for pre-specified confounding variables noted in Fig. 2 of the trial protocol [22].

^c^*RD* risk difference. RD calculated from marginal probabilities. Confidence intervals were calculated using a pairwise comparison of margins after fitting a GEE model using Stata, allowing for clustering of observations within practices

^d^The Knowledge variable was coded as indicating inadequate (0) or adequate (1) knowledge about key messages of the guideline

For the clinical behaviour ‘advising patients to stay active’, there was a statistically significant difference between groups in *Intention (generalised and performance)*, *Social influences (direct)*, and *Memory*. This demonstrates clinicians in the intervention group had greater intention to advise patients to stay active, perceived greater social pressure to provide advice to stay active, and were more likely to remember to give this advice than clinicians in the comparator group. Clinicians in the intervention group also demonstrated lower fear-avoidance beliefs about physical activity at 3 months (Adj effect − 1.63, 95% CI − 2.81 to − 0.45; scale range 0–24).

### Cost-effectiveness of the intervention

Additional File, Table 3 provides an overview of the ALIGN intervention components, including mode of delivery, provider, recipient, timing, and intensity. The direct costs associated with delivery of the ALIGN intervention components are additional to costs associated with the existing guideline dissemination strategy. Results from the main effectiveness analysis suggest that hypothesised cost-savings due to reductions in health service utilisation and/or productivity gains are unlikely to offset the direct costs of ALIGN intervention components.

With respect to hypothesised reductions in utilisation of X-ray and imaging, there was no statistically significant treatment effect on X-ray referral as measured by the clinician-completed checklist (Adj OR: 1.40; 95% CI 0.51, 3.87), X-ray occurred to 3-month follow-up as measured by patient self-report (Adj OR: 0.74; 95% CI 0.38, 1.41), imaging referral as measured by clinician completed-checklist (Adj OR: 0.87; 95% CI 0.30, 2.53), or imaging referral as measured by file audit (Adj OR: 1.47; 95% CI 0.54, 4.01). For the 428 out of 1358 (32%) patients in whom file audit could be completed, we found a significantly lower rate of X-ray referral in the intervention group than in the comparison group (Adj OR: 0.23; 95% 0.07, 0.72). While this result is consistent with a similar reduction in *intention* for X-ray referral (Adj OR 0.27; 95% CI 0.17, 0.44), the potential for selection to have biased results in our small sample of file audit patients and the absence of a treatment effect in favour of the intervention group on the primary outcome suggest that cost-offsets due to lower rates of X-ray and imaging referral are unlikely to materialise.

With respect to the potential for broader cost-offsets, improvements in giving advice to stay active (Adj OR: 1.96; 95% CI 1.20, 3.22; Adj RD: 0.10; 95% CI 0.01, 0.19) and intention to give advice to stay active: Adj OR: 2.37; 95% CI 1.51, 3.74) did not translate into clinically important gains in final outcomes of interest. Specifically, differences in low back pain-specific disability (Adj mean difference: 0.37; 95% CI 0.48, 1.21) and pain severity (Adj mean difference: − 0.10; 95% CI − 0.48, 0.28) were neither statistically nor clinically significant. Results from the main effectiveness analysis are therefore not consistent with a causal effect on health service utilisation or productivity gains via accelerated recovery and improved patient health outcomes. These results suggest that we can exclude the possibility that ALIGN is cost-saving *without proceeding to a full economic evaluation.*

Primary and secondary outcomes for the economic evaluation were selected to measure two potential sources of health benefit: mortality and morbidity effects beyond trial-end due to reduced exposure to ionising radiation (i.e. proxied by rates of X-ray and imaging referral) and improvements in low back pain-specific disability and health-related quality of life due to accelerated recovery. Results for X-ray and imaging referral from the main effectiveness analysis (reported above) are not consistent with reduced exposure to ionising radiation. Results are similarly unsupportive of a health gain for low back pain-specific disability (Adj mean difference: 0.37; 95% CI 0.48, 1.21), health-related quality of life at 3 month follow-up (Adj mean difference: − 0.027; 95% CI − 0.069, 0.015), and quality-adjusted life-years to 3-month follow-up (Adj mean difference: − 0.004; 95% CI − 0.012, 0.003).

## Discussion

### Summary of findings

We evaluated effectiveness of a theory-informed implementation intervention that aimed to improve chiropractors’ and physiotherapists’ adherence to recommendations from a clinical practice guideline for acute low back pain. The intervention led to changes in outcomes proximal to the intervention (i.e. predictors of clinician behaviour and intention) but did not lead to important changes in the practitioner behaviour of X-ray referral, nor patient health outcomes, between the group of practitioners who underwent the intervention and the comparator group. There were some exceptions to this, with patients in the intervention group being more likely to receive advice to stay active than those in the comparator group, and intervention group clinicians having greater intention to advise patients to stay active and to manage without plain X-ray than comparator group clinicians. Differences between groups in some of the other hypothesised predictors of clinician behaviour in favour of the intervention were also found (e.g. beliefs about capabilities, social influences).

In our study, a single educational event, albeit one underpinned with behaviour change theory and complemented by academic detailing, did not lead to meaningful practice change. Moreover, the ALIGN intervention is likely to impose net costs on the health system. Taken together, these results suggest that the ALIGN intervention is unlikely on its own to represent a cost-effective use of resources. Our results are consistent with other trials of these types of implementation interventions in that clinician practice behaviour appears resistant to change, and we do not yet have a clear answer as to which interventions increase the uptake of evidence in these contexts [19]. In fact, our group conducted a similar trial with general medical practitioners that found similar results [47, 48]. Education may be necessary, but it does not seem sufficient for changing complex practice behaviours [49].

We provide detail on the theoretical basis, delivery, and measures of the process of care that we targeted in our intervention. When choosing our intervention components, we considered theory, evidence, and practical considerations [30]. This led us to choose an educational intervention, comprising a series of BCTs delivered as part of a symposium. The symposium was a delivery mode that clinicians were familiar with, and we used this as the ‘packaging’ to deliver the BCTs, as opposed to education comprising simple information provision. We explicitly considered barriers to change and mechanisms of action of the implementation intervention, underpinned by a theoretical framework that attempted to explain how and why the intervention may have effected change. Even with these procedures in place, we were unable to demonstrate meaningful practice change.

### Possible explanations for findings

When planning ALIGN, we decided to use multiple methods to measure the primary outcome of whether a practitioner ordered or undertook an X-ray, because we could not identify a single optimal measurement method. The clinician-completed checklist, including X-ray use (primary outcome), only captured data for a 2-week period, and was self-reported by clinicians. Patient recall about whether or not they were referred for X-ray as documented on the patient-completed questionnaire also has limitations, and the timing of the X-ray received may not have been related to their current episode of low back pain. The file audit was resource intensive and required an additional level of consent from patients; hence, we were only able to undertake this in a limited number of practices (54%) and for a subset of patients (32%). In addition, the measurement periods were different, with the clinician-completed checklist capturing only a 2-week period in a patient’s episode, while the file audit and patient questionnaire intended to capture whether an X-ray occurred for that episode of acute low back pain. As expected, this led to different prevalence of X-ray referral in the intervention and comparator groups, and their contrasts, across the measurement methods. Given there was no optimal method, the results are complicated to interpret. Ideally, clinical administrative data would be available for all participants to measure this outcome of X-ray referral behaviour.

The percentage of patients referred for X-ray was low in both the intervention and the comparator group, regardless of the method of measurement. While it may be that the intervention itself was not sufficient to lead to meaningful change in X-ray referral, other explanations are possible. For example, the comparator group rate of X-ray referral measured by the checklist was low at only 3%, compared to our anticipated 37% used in the sample size calculation. For the clinicians in this study, the evidence-to-practice gap for X-ray was smaller than we had predicted. It may be that the evidence-to-practice gap with respect to X-ray use in physiotherapy and chiropractic practice was much smaller than anticipated, or that the clinicians in the trial who agreed to participate were a non-representative group who were more likely to adhere to the guideline recommendations about imaging than other clinicians who did not participate. Also, clinicians in this study were aware that their self-report was being assessed, so it is possible that they practised in-line with guidelines for the two-week data collection period, knowing that their responses were contributing to the study. These latter explanations seem likely, with rates of imaging for low back pain remaining high (approximately 25%) and unchanged in the primary care setting over the last two decades [50], where inappropriate imaging is common [51]. Therefore, with a lower than expected X-ray referral rate and most clinicians appearing to practise in-line with the guideline, there was limited ability to bring about any meaningful practice change.

When measured by questionnaire, we saw important differences between groups in regard to clinicians’ intention to undertake both targeted evidence-based clinical practice behaviours (manage without X-ray and give advice to stay active) and some other hypothesised predictors (e.g. *Beliefs about capabilities*, *Beliefs about consequences*, *Professional role and identity*, and *Social influences*) for managing without X-ray. However, this did not translate to actual clinician behaviour change for the X-ray behaviour. This may demonstrate the substantial challenges faced in changing behaviour with this type of one-off intervention event, even if clinician intention is changed. Alternately, there may be other factors that could regulate behaviour change but were not addressed by this intervention, e.g. having X-ray facilities readily accessible on site or via bulk-billing radiology services (related to TDF domain *Environmental context and resources*), clinician fear of missing underlying sinister pathology or litigation for misdiagnosis (related to TDF domain *Emotion*), as well as entrenched habits (e.g. use of imaging as first line diagnostic tool). That we were unable to demonstrate change in clinical practice despite improvement in intention to practice according to the two recommendations may also be explained by a number of limitations in our study.

We measured intention using patient vignettes, which may not have accurately reflected actual patient scenarios and so potentially overestimated intention. A systematic review of 10 studies (including 1623 health professionals) has explored the relationship between intention and clinical behaviour [52]. This review included five studies in which, like ours, clinician behaviour was measured by self-report. The review found high correlation between intention and self-reported clinical behaviour, which is inconsistent with our results. In addition, there were differences in setting for studies included in the review in that none related to imaging or chiropractic and physiotherapy practice.

Fidelity of intervention delivery in this trial was poor with only 58% of clinicians in the intervention group actually attending the symposium and so receiving the main component of the intervention. In addition, only 35% reported watching the DVD, and 66% received a follow-up call. Overall, only about half of those in the intervention group actually received the full intervention. In addition, for those who did attend, some symposium elements were not delivered as planned (e.g. skills demonstration session, small group practical, straw poll, reflective activity). This lack of engagement with the intervention could have led to a smaller effect. Fidelity findings show that several of the BCTs that were part of the implementation intervention as designed were not actually delivered. Notably, BCTs that support the translation of intentions into behaviour (skills demonstration and behavioural rehearsal) were not delivered with high fidelity. This could explain the observed intention-behaviour discrepancy. The possibility that low fidelity influenced the results is supported by the observation that fidelity was higher in the symposium for chiropractors than for the physiotherapist sample, while the effects of the intervention were greater for the chiropractor group than for the physiotherapist group.

Although the main recommendations in the guideline we implemented in this study are still current, that is avoiding routine imaging and giving advice to stay active, more recent low back pain guidelines have an increased focus on self-management, addressing psychosocial issues, and stratified care [16–18]. Implementation of more recent guideline recommendations for the management of low back pain likely require more complex models of care [2, 53] and may need different tailored implementation approaches to those we tested [54, 55].

Many implementation studies do not measure patient outcomes since the clinical behaviours or actions to be implemented have already been shown through research to improve patient outcomes [56]. Like in our previous study in general medical practice setting [47, 57], we chose to measure patient outcomes since it was not clear that a change in clinician behaviour would result in a change in the patient’s health status. Also, the trials that underpin the key message from the guideline on providing advice to stay active have used different interventions, delivered in different ways, have included different comparator arms, and have only shown small beneficial effects for the outcomes pain, rate of recovery, and function. We demonstrated no important difference between the groups despite patients in the intervention group being more likely to have received advice to stay active from their treating clinician than patients in the comparator group. This may be due to inadequacies in the advice given, different ways of delivering the advice, or patients not following the advice. Importantly, only 10% more patients in the intervention group received advice to stay active, making it difficult to observe a difference in patient outcomes, even if there had been an improvement for this group. What may have been a more relevant measure for patient outcomes was a self-reported, or objective measure, of physical activity level; future studies should consider this outcome when this key guideline message is being implemented.

### Strengths and limitations

This was a large cluster randomised trial, with blinded outcome assessors, and blinded statistician who undertook the data analysis. There are a number of study limitations.

We stratified our randomisation based on practice location (rural or metropolitan) and practitioner type (chiropractor or physiotherapist); however, there were some between group differences at baseline. Intervention group practices were more likely to have an X-ray facility on site and were more likely to have access to a bulk-billing radiology service, and clinicians within these practices indicated lower intention to adhere to guidelines at baseline. For X-ray and imaging referral measured via the checklist, our estimates of the intervention effect may therefore be biased due to confounding arising from these differences. For these outcomes, we were unable to adjust for pre-specified baseline potential confounders (including X-ray facility, access to a bulk-billing radiology service, and intention to adhere to the guidelines), due to limited non-adherence events; however, for X-ray and imaging referral measured via file audit, we were able to adjust for these potential confounders, limiting bias in these estimates.

Although we enrolled more clinicians than anticipated, they subsequently enrolled fewer patients during the 2-week data collection period than our determined sample size (449 patients enrolled versus 2720 planned). Although we enrolled more clinicians than anticipated, they subsequently enrolled fewer patients during the 2-week data collection period than our determined sample size (449 patients enrolled versus 2720 planned). For the primary clinician outcome X-ray referral, on which the sample size was determined, the confidence interval for the risk difference was narrow. This was due to the observed X-ray referral rates being substantially smaller than those assumed for the sample size calculation, thus allowing us to conclude with certainty for this outcome. Further, because providers were unblinded to treatment assignment there was potential to selectively enrol patients; however, we directed clinicians to enrol consecutive patients during the data collection period in an attempt to mitigate such selection bias. The characteristics of recruited patients across the intervention and comparison groups were similar (Table 4), indicating no obvious differential recruitment of patients.

While our intention-to-treat analysis provides estimates of the ‘effect of assignment to the intervention’ [58], and therefore provides unbiased estimates of the effect of assignment, if it were to be rolled out in the real-world, an analysis that attempts to estimate the ‘effect of adhering to the intervention’ may yield different estimates; however, we believe the pertinent effect of interest is the former. In order to facilitate intervention fidelity if other researchers choose to deploy a similar implementation intervention, the Additional File, Table 3 gives a comprehensive overview of ALIGN intervention components for other investigators to use as a guide.

## Conclusions

The implementation intervention comprising a single educational event for chiropractors and physiotherapists, and including specified BCTs, did not lead to meaningful change between groups in the primary study outcomes, X-ray referral behaviour, and patient outcomes. The intervention did lead to an improvement for giving advice to stay active and intending to adhere to the guideline recommendations regarding referral for X-ray. A number of limitations in the conduct of this study, including a floor effect for X-ray referral, smaller patient sample size than planned, low intervention fidelity, and measurement challenges, mean we are not able to draw firm conclusions about the effect of this implementation intervention. What is clear is that delivery of the ALIGN intervention comes at a cost. Without convincing evidence of cost-offsets due to reduced healthcare utilisation or productivity gains or improvements in patient health outcomes, we cannot recommend the ALIGN intervention as a cost-effective use of resources.

## Supplementary Information

**Additional file 1: Table 1.** Deviations from trial protocol. **Table 2.** Labels used to describe outcomes across the trial report, protocol and registry entry. **Table 3.** Overview of ALIGN intervention components. **Table 4.** Baseline values for hypothesised predictors of clinician behaviour –all clinicians

## Data Availability

The datasets used and/or analysed during the current study are available from the corresponding author on reasonable request.
