# Supplementary material for: Improving adherence to acute low back pain guideline recommendations with chiropractors and physiotherapists: the ALIGN cluster randomised controlled trial
Source: Trials. 2022 Feb 14;23:142. doi: 10.1186/s13063-022-06053-x (PMC8842895; doi:10.1186/s13063-022-06053-x)
Supplement: Supplementary file 1 — Additional file 1: Table 1. Deviations from trial protocol. Table 2. Labels used to describe outcomes across the trial report, protocol and registry entry. Table 3. Overview of ALIGN intervention components. Table 4. Baseline values for hypothesised predictors of clinician behaviour –all clinicians [file 13063_2022_6053_MOESM1_ESM.pdf]

Additional File

Explanation of retrospective registration of the ALIGN cluster randomized trial

The ALIGN trial was labelled as 'retrospective registration' by the Australian New Zealand Clinical Trials Registry (https://www.anzctr.org.au/Trial/Registration/TrialReview.aspx?id=320855); however, the time between the submission of the registration and enrolment of the first cluster (physiotherapy/chiropractic clinic) occurred within two days. Recruitment of the patient participants to the clusters did not take place until approximately 6 months post trial registration.

A timeline of activities in the trial follows:

- 4/11/2009: ethics approval received*
- 18/11/2009: first cluster enrolled*
- 20/11/2009: Australian New Zealand Clinical Trials Registry submission date*
- 25/11/2009: trial registered*
- 20/01/2010: clusters randomised
- 20-27/02/2010: main intervention delivered to clusters
- May 2010: first patient participant enrolled

* Dates noted in the registry entry.

The trial design details were submitted, and approved by, the Registry, well before patient participants were enrolled in the study, or before any outcome data were collected.

Additional File, Table 1. Deviations from trial protocol

Summary	Plan as per trial protocol	Deviation from protocol	Justification for deviation
Change to clinical file audit sample	We planned to undertake the clinical file audit for all consenting patients	We undertook the clinical file audit for consenting patients at urban practices only	This change was due resources constraints
Description of secondary outcomes	Measurement of secondary outcomes intention and behavioural constructs	None	In the trial registry entry, the secondary outcomes "intention and behavioural constructs" were described in only general terms. The published trial protocol gave full details of these secondary outcomes (Table: "Details of the outcome measurement for the behavioural constructs", Additional file 3, pages 7-9), and we report these outcomes in the trial report
Analysis section: change to adjustment of confounders	For each outcome, we pre-specified stratification variables and confounders that would be adjusted for (Figure 2 trial protocol (22)). In the circumstance where there was limited data or events, or both, we planned to adjust for only the stratification variables and baseline of the outcome (where appropriate)	1) For the outcomes 'X-ray referral' and 'Imaging referral' (measured by the clinician checklist), we only adjusted for the stratification variables<br>2) For the outcome 'Advised bed rest' and the subgroup analysis of 'X-ray referral' (measured by the clinician checklist), we did not adjust for stratification or pre-specified confounders<br>3) For the outcomes 'LBP specific disability' (patient questionnaire), 'Pain severity' (patient questionnaire), 'X-ray occurred' (patient questionnaire), 'Fear-avoidance	1) and 2): These changes were due to a limited number of events<br><br>3): These potential confounding variables were to be extracted from the clinical file audit. However, given the file audit was restricted to urban practices, this led to missing information for these variables for patients in rural practices

		beliefs' (patient questionnaire), 'X-ray referral' (file audit), 'Imaging referral' (file audit), we did not adjust for two pre-specified confounders 'No. visits for this episode of acute LBP' and '≥ 1 x-ray LBP previous 12 mths'	
Analysis section: change of method	We had planned to calculate risk differences from the GEEs using an identity link function in place of the logit link function	We used marginal standardisation. Estimated regression coefficients from the fitted GEEs (with logit link functions, robust variance estimation) were used to calculate average predicted proportions with the outcome in each group. Risk differences were calculated from these proportions.	GEEs with an identity link function and adjustment for covariates can suffer from convergence issues
Analysis section: change to missing data analysis	We had planned to investigate methods to impute missing measures of outcomes at baseline (e.g. baseline predictors of clinician behaviour)	We did not impute missing measures of outcomes at baseline	There was a small amount of missing data at baseline
Cost-effectiveness analysis	We had planned to undertake an economic evaluation with the aim of quantifying additional costs (savings) and health gains arising from the ALIGN intervention as compared to access to the guideline via existing practice	Cost-consequence analysis presented	Simplify analysis and interpretation of results

Additional File, Table 2. Labels used to describe outcomes across the trial report, protocol and registry entry

Trial report outcome labels[a]					Protocol outcome labels[b]	Registry entry outcome labels[c]	
**Outcome** ***(outcome category)***	**Data collection method**	**Outcome assessment period**	**Source**	**Level at which data collected**		**ANZCTR label**	**Trial registry [outcome label] description****
**Inference intended at the clinician level**							
X-ray referral *(primary outcome, clinician behaviour)*	Checklist completed by clinician	3 - 4 mths (months) post symposium	Clinician	Patient	X-ray referral *(practitioner behaviour)*<br><br>3 - 4 mths post symposium	Primary outcome [1]	*Primary outcome [1]*: **X-ray referral (measured via practitioner-reported** encounter forms with consecutive patients who have acute non-specific low back pain) *Timepoint [1]*: Encounter forms are completed immediately following patient consultations during a two-week data collection period. Data collection period occurs 3-4 months after intervention delivery.
Advice to stay active *(clinician behaviour)*	Checklist completed by clinician	3 – 4 mths post symposium	Clinician	Patient	Advice to stay active *(practitioner behaviour)*<br><br>3 - 4 mths post symposium	Secondary outcome [1]	*Secondary outcome [1]:* **Advice to stay active (measured via practitioner-reported** encounter forms with consecutive patients who have acute non-specific low back pain) *Timepoint [1]:* Encounter forms are completed immediately following patient consultations during a two-week data collection period. Data collection period occurs 3-4 months after intervention delivery.
Imaging referral excluding X-ray *(clinician behaviour)*	Checklist completed by clinician	3 – 4 mths post symposium	Clinician	Patient	Imaging referral excluding x-ray *(practitioner behaviour)*<br><br>3 - 4 mths post symposium	Secondary outcome [3]	*Secondary outcome [3]:* **Any imaging referral (measured via practitioner-reported** encounter forms with consecutive patients who have acute non-specific low back pain) *Timepoint [3]:* Encounter forms are completed immediately following patient consultations during a two-week data collection period. Data collection period occurs 3-4 months after intervention delivery.

Advised bed rest *(clinician behaviour)*	Checklist completed by clinician	3 – 4 mths post symposium	Clinician	Patient	Advised bed rest *(practitioner behaviour)*<br><br>3 - 4 mths post symposium	Secondary outcome [2]	*Secondary outcome [2]:* **Advised bed rest (measured via practitioner-reported** encounter forms with consecutive patients who have acute non-specific low back pain)<br>*Timepoint [2]:* Encounter forms are completed immediately following patient consultations during a two-week data collection period. Data collection period occurs 3-4 months after intervention delivery.
X-ray referral (file audit) *(clinician behaviour)*	Clinical file audit	0 – 7 mths post symposium	Clinician case notes	Patient	X-ray referral (file audit) *(practitioner behaviour)*<br><br>0 - 7 mths post symposium	Secondary outcome [11]	*Secondary outcome [11]:* **X-ray referral (measured via files** of consecutive patients who have acute non-specific low back pain who consent to file audit).<br>*Timepoint [11]:* Audit of patient files will be done at 7 months following intervention/control delivery.
Imaging referral excluding X-ray (file audit) *(clinician behaviour)*	Clinical file audit	0 – 7 mths post symposium	Clinician case notes	Patient	Imaging referral excluding x-ray (file audit) *(practitioner behaviour)*<br><br>0 - 7 mths post symposium	Secondary outcome [12]	*Secondary outcome [12]:* **Imaging referral excluding x-ray (measured via files** of consecutive patients who have acute non-specific low back pain who consent to file audit).<br>*Timepoint [12]:* Audit of patient files will be done at 7 months following intervention/control delivery.
Intention to adhere to guideline recommendations: X-ray referral; Imaging referral excluding X-ray; Advice to stay active; Bed rest advice *(predictors of clinician behaviour)*	Questionnaire	Baseline, 4 mths post symposium	Clinician	Clinician	Intention to adhere to CPG recommendations: X-ray referral; Imaging referral excluding x-ray; Advice to stay active; Bed rest advice *(predictor practitioner behaviour)*<br><br>Baseline, 4 mths post symposium	Secondary outcome [4]	*Secondary outcome [4]:* **Practitioner behavioural constructs** (attitudes, beliefs and intentions) measured via questionnaire<br>*Timepoint [4]:* At baseline and 4 months following intervention/control delivery.

Beliefs about capabilities, beliefs about consequences, knowledge, professional role and identity, social influences, environmental context and resources, memory[c] *(predictors of clinician behaviour)*	Questionnaire	Baseline, 4 mths post symposium	Clinician	Clinician	Behavioural constructs (e.g., knowledge, beliefs about capabilities)[d] *(predictor practitioner behaviour)*<br><br>Baseline, 4 mths post symposium	Secondary outcome [4]<br><br>Secondary outcome [5]	*Secondary outcome [4]:* **Practitioner behavioural constructs** (attitudes, beliefs and intentions) measured via questionnaire *Timepoint [4]:* At baseline and 4 months following intervention/control delivery.<br><br>Secondary outcome [5]: **Practitioner fear avoidance beliefs** (measured via questionnaire) *Timepoint [5]:* At baseline and 4 months following intervention/control delivery.
**Inference intended at the patient level**							
LBP specific disability *(primary outcome, health outcome)*	Questionnaire	3 mths post onset acute LBP episode	Patient	Patient	LBP specific disability *(health outcome)*<br><br>3 mths post onset acute LBP episode	Primary outcome [2]	*Primary outcome [2]:* **Low-back pain specific disability** (measured via Roland-Morris Disability Questionnaire [Roland M, Morris R. A study of the natural history of back pain. Part I: development of a reliable and sensitive measure of disability in low-back pain. Spine 1983;8:141-4]) *Timepoint [2]:* Questionnaires will be completed by patients 3 months post-onset of acute low-back pain episode
Pain severity *(health outcome)*	Questionnaire	3 mths post onset acute LBP episode	Patient	Patient	Pain severity *(health outcome)*<br><br>3 mths post onset acute LBP episode	Secondary outcome [6]	*Secondary outcome [6]:* **Patient usual pain** (measured via questionnaire using an 11-point numerical rating scale (0-10)) *Timepoint [6]:* At 3 months after onset of acute low-back pain episode.
X-ray occurred *(health behaviour)*	Questionnaire	3 mths post onset acute LBP episode	Patient	Patient	X-ray occurred *(health behaviour)*<br><br>3 mths post onset acute LBP episode	Secondary outcome [7]	*Secondary outcome [7]:* **Patient reports referral for an x-ray or received an x-ray** from their chiropractor or physiotherapist for the current episode of low-back pain (measured via questionnaire) *Timepoint [7]:* At 3 months after onset of acute low-back pain episode
Fear-avoidance beliefs *(predictor health behaviour)*	Questionnaire	3 mths post onset acute LBP episode	Patient	Patient	Fear-avoidance beliefs *(predictor health behaviour)*<br><br>3 mths post onset acute LBP episode	Secondary outcome [8]	*Secondary outcome [8]:* **Patient fear avoidance beliefs** (measured via Fear Avoidance Beliefs Questionnaire [Waddell G, Newton M, Henderson I, et al. A Fear-Avoidance Beliefs Questionnaire (FABQ) and the role of fear-avoidance beliefs in chronic

							low back pain and disability. Pain 1993;52:157-68]) *Timepoint [8]:* At 3 months after onset of acute low-back pain episode.
Health-related Quality of Life *(health outcome)*	Questionnaire	3 mths post onset acute LBP episode	Patient	Patient	Health-related Quality of Life *(health outcome)*<br><br>3 mths post onset acute LBP episode	Secondary outcome [9]	*Secondary outcome [9]:* **Patient quality of life** (measured via Assessment of Quality of Life Questionnaire [Hawthorne G, Richardson J & Osborne R. The assessment of Quality of Life (AQoL) instrument: a psychometric measure of health-related quality of life. Quality of Life Research 1999;8:209-224]) *Timepoint [9]:* At 3 months after onset of acute low-back pain episode.
Health Service Utilisation and Productivity Gains/Losses	Questionnaire	3 mths post onset acute LBP episode	Patient	Patient	Health Service Utilisation and Productivity Gains/Losses<br><br>3 mths post onset acute LBP episode	Secondary outcome [10]	*Secondary outcome [10]:* **Health service utilisation used by patient** (measured via Health Service Utilisation items in questionnaire) *Timepoint [10]:* At 3 months after onset of acute low-back pain episode.

[a] Table 1 from the trial report.

[b] Outcome label from the trial protocol (Table 1, (32))

[c] Outcome labels in the Australia New Zealand Clinical Trials registry entry (https://www.anzctr.org.au/Trial/Registration/TrialReview.aspx?id=320855)

[d] Further detail of the behavioural construct domains is available in Table 2 and the table "Details of the outcome measurement for the behavioural constructs" in additional file #3 of the protocol (32).

Additional File, Table 3. Overview of ALIGN intervention components

	Symposium	Written educational material	DVD	Academic detailing
**Intervention content**	Interactive symposium-style event comprising the following elements:<br>1. Keynote speech relevant to the guideline recommendations delivered by clinical opinion leader<br>2. Video-recordings reinforcing guideline messages from clinical opinion leaders and consumer advocate<br>3. Small group discussion led by trained clinical facilitators<br>4. Skills demonstration sessions by clinical opinion leader<br>5. Small group practical with simulated patients (trained actors) led by trained clinical facilitators<br>6. Audience straw polling<br>7. Reflective activity<br><br>Incorporating behaviour change techniques (BCTs):<br>1. *Increasing skills* - providing an opportunity to increase the skills needed to perform a particular behaviour by presenting scenarios of varying difficulty;<br>2. *Rehearsal of relevant skills* - providing an opportunity to practice how to correctly perform a behaviour;<br>3. *Social processes of encouragement, pressure and support* - utilising peers to encourage, pressure, and/or support an individual to adopt a proposed behaviour change; facilitate discussion or observation of peers' performance; and provide opportunities for social comparison;<br>4. *Feedback* - providing an evaluation of one's performance in relation to a set standard or others' performance;<br>5. *Persuasive communication* - verbal and non-verbal techniques employed to convince a person the strengths of one argument/viewpoint over another (e.g., use of a credible source to deliver information; emphasising the quality of evidence underpinning a recommendation; appealing to emotion e.g., fear, guilt; refuting opposing arguments);	Supporting materials comprising:<br>1. Symposium program and presentation handouts<br>2. Reflective activity instructions<br>3. Copy of the guideline for managing acute low back pain<br>4. Clinical algorithm for managing acute low back pain (based on guideline)<br>5. Patient information sheet on 'What is acute low back pain?'<br><br>Relevant BCT:<br>1. *Information provision*.<br><br>Selected to alter or redirect *Knowledge.*	Video recording of symposium didactic sessions by clinical opinion leaders<br><br>Relevant BCTs:<br>1. *Social processes of encouragement, pressure and support*;<br>*2. Persuasive communication;*<br>*3. Information provision;*<br>*4. Information regarding behaviour, outcome.*<br><br>Selected to alter or redirect<br>*1. Beliefs about capabilities;*<br>*2. Beliefs about consequences;*<br>*3. Knowledge;*<br>*4. Professional role and identity.*	A scheduled follow-up telephone call to discuss difficulties encountered in implementing the guideline recommendations and strategies to overcome these<br><br>Relevant BCTs:<br>*1. Social processes of encouragement, pressure and support*<br>2. *Persuasive communication*<br>*3. Information provision*<br>4. *Information regarding behaviour, outcome*<br><br>Selected to alter or redirect<br>*1. Beliefs about capabilities;*<br>*2. Beliefs about consequences;*<br>*3. Knowledge;*

	6. *Information regarding behaviour and outcome* - providing information about a behaviour-health link (e.g., linking better health outcomes with performance of the target behaviour), about consequences (e.g., information about the benefits and costs of action or inaction, focusing on what will happen if the person does or does not perform the behaviour), and about others' approval (e.g., information about what others think about the persons' behaviour and whether others will approve or disapprove of any proposed behaviour change);<br>7. *Modelling* - an expert/credible model shows how to correctly perform a behaviour;<br>8. *Information provision* - providing general information (e.g., information about the evidence underlining a particular recommendation).<br><br>BCTs selected to alter or redirect the following hypothesised determinants of clinician behaviour:<br>1. *Beliefs about capabilities* – the extent to which the clinician feels confident in/control over performing the behaviour;<br>2. *Beliefs about consequences* – the extent to which the clinician is in favour of performing the behaviour and has positive behavioural beliefs;<br>3. *Knowledge* – whether the clinician has knowledge of the behaviour;<br>4. *Professional role and identity* – the extent to which the clinician feels it is their professional responsibility to perform the behaviour;<br>5. *Social influences* – the extent to which the clinician feels social pressure to engage in the behaviour;<br>6. *Intention* – the extent to which the clinician intends to perform the behaviour			*4. Professional role and identity.*
**Who provided**	• Senior research team clinicians<br>• Clinical opinion leaders (physiotherapists/chiropractors identified in consultation with representatives from their respective professional associations<br>• Radiologist<br>• Consumer advocate	Senior research team clinicians and clinical opinion leaders	Clinical opinion leaders	Senior research team clinicians

**Who received**	Clinicians in the intervention group attending the symposium. Separate symposia held for physiotherapists and chiropractors	All clinicians in the intervention group, including those who were not able to attend the symposium	All clinicians in the intervention group, including those who were not able to attend the symposium	Clinicians in the intervention group, including those who were not able to attend the symposium, who were available for follow-up contact
**Mode of delivery**	Face-to-face, including large group didactic sessions and small group interactive sessions	In hardcopy	By post	By telephone
**Setting**	University-based conference venue	At symposium venue and posted to clinicians workplace address	Clinicians workplace address	At clinicians workplace
**When and how much**	One-full day (8.5hr) symposium held on 20 February 2010 for physiotherapists and 27 February 2010 for chiropractors	At symposium and 4 weeks following symposium by post	4 weeks following symposium	One follow-up telephone contact of 10-15 min duration at 2-4 weeks following symposium attendance or receipt of DVD
**Tailoring**	Content tailored to overcome the modifiable barriers and enhance the enablers identified by physiotherapists and chiropractors in prior semi-structured interview and survey studies. No further tailoring to participants was undertaken	Content tailored to overcome the modifiable barriers and enhance the enablers identified by physiotherapists and chiropractors in prior semi-structured interview and survey studies. No further tailoring to participants was undertaken	See symposium	Content tailored to clinician's difficulties in implementing behaviour change, and possible strategies to overcome these difficulties
**Modifications**	No modifications occurred	No modifications occurred	No modifications occurred	No modifications occurred
**Fidelity**	58% (74/104) of clinicians in the intervention group (46/85 physiotherapists and 28/43 chiropractors) attended the symposium.	58% (74/104) of clinicians in the intervention group received the educational	All clinicians in the intervention group were sent the DVD. 35% (45/104) of	66% (85/104) of clinicians in the intervention group

	An independent assessor recorded delivery, as planned, of 43% (3/7) of intervention elements and 57% (21/37) of BCTs at the physiotherapy symposium. Symposium elements not delivered as planned were:<br>1. Skills demonstration session on effectively communicating with patients and giving advice to stay active - not done<br>2. Small group practical to rehearse diagnostic and communication skills with simulated patients - modified; some participants didn't rehearse relevant skills; some facilitators didn't model target behaviours; less time spent on small group practical than planned<br>3. Straw poll at end of day - modified; included fewer questions than planned<br>4. Reflective activity - not done.<br>The independent assessor at the chiropractic symposium recorded delivery of 86% (6/7) of intervention elements and 76% (28/37) of BCTs as planned. Symposium elements not delivered as planned were:<br>1. Small group practical to rehearse diagnostic and communication skills with simulated patients - modified; less time spent on small group practical than planned.	materials via attendance at the symposium.<br>All clinicians in the intervention group were sent the written educational materials four weeks following the symposium.	clinicians in the intervention group reported viewing the DVD.	received the follow-up telephone call.

Additional File, Table 4. Baseline values for hypothesised predictors of clinician behaviour – all clinicians

		Comparison group				Intervention group			
**Variable**	**Value range[a]**	**No. practices**	**No. clinicians**	**N (%) / mean (sd)**	**Median (IQR)**	**No. practices**	**No. clinicians**	**N (%) / mean (sd)**	**Median (IQR)**
**Managing patients without referral for plain X-ray**									
Intention (generalised)	1 to 7	97	129	5.5 (1.45)	6.0 (4.7 to 6.7)	95	115	5.3 (1.75)	6.0 (4.7 to 6.7)
Intention (performance)	0 to 10	96	128	2.1 (2.95)	1.0 (0.0 to 3.0)	93	111	2.7 (3.28)	1.0 (0.0 to 4.0)
Beliefs about capabilities	1 to 7	97	129	5.6 (1.13)	5.8 (4.8 to 6.5)	95	115	5.6 (1.07)	6.0 (5.0 to 6.5)
Beliefs about consequences (direct)	1 to 7	96	128	5.4 (1.27)	5.6 (4.5 to 6.4)	92	112	5.3 (1.53)	5.8 (4.4 to 6.4)
Beliefs about consequences (behavioural beliefs)	1 to 7	96	128	4.8 (1.01)	5.0 (4.3 to 5.6)	93	113	4.7 (1.11)	4.9 (4.0 to 5.6)
Knowledge[b]	0 or 1	97	129	6 (5%)		94	114	3 (3%)	
Professional role and identity	1 to 7	96	126	4.5 (1.09)	4.8 (3.8 to 5.2)	95	114	4.5 (1.21)	4.6 (4.0 to 5.4)
Social influences (direct)	1 to 7	97	129	4.9 (1.33)	5.0 (4.3 to 6.0)	95	115	4.9 (1.58)	5.0 (4.0 to 6.0)
Social influences (indirect)	1 to 7	97	129	4.7 (0.95)	4.8 (4.3 to 5.3)	95	115	4.8 (0.99)	5.0 (4.0 to 5.5)
Environmental context and resources	1 to 7	97	129	4.1 (2.05)	4.0 (2.0 to 6.0)	94	114	3.6 (2.10)	4.0 (2.0 to 5.0)
**Advising patients to stay active**									
Intention (generalised)	1 to 7	97	129	6.2 (0.93)	6.3 (5.7 to 7.0)	95	115	6.1 (1.08)	6.3 (5.7 to 7.0)
Intention (performance)	0 to 10	96	128	8.9 (1.65)	10.0 (8.0 to 10.0)	93	112	8.7 (1.86)	10.0 (8.0 to 10.0)
Beliefs about capabilities	1 to 7	97	129	5.9 (0.84)	6.0 (5.3 to 6.5)	95	115	6.1 (0.75)	6.3 (5.8 to 6.8)
Beliefs about consequences (direct)	1 to 7	97	128	6.2 (1.01)	6.6 (6.0 to 7.0)	94	113	6.2 (1.20)	6.6 (6.0 to 7.0)
Beliefs about consequences (behavioural beliefs)	1 to 7	96	127	5.3 (0.65)	5.3 (5.0 to 5.7)	94	114	5.2 (0.68)	5.3 (4.9 to 5.7)
Fear-avoidance beliefs	0 to 24	95	127	10.7 (4.89)	11.0 (8.0 to 14.0)	95	115	11.7 (5.20)	11.0 (8.0 to 16.0)
Knowledge[b]	0 or 1	97	129	110 (85%)		95	115	86 (75%)	
Professional role and identity	1 to 7	96	128	6.4 (0.76)	6.7 (6.0 to 7.0)	94	114	6.5 (0.84)	6.7 (6.3 to 7.0)
Social influences (direct)	1 to 7	97	129	5.7 (1.06)	6.0 (5.0 to 6.7)	95	115	5.8 (1.17)	6.0 (5.3 to 6.7)
Social influences (indirect)	1 to 7	95	127	5.3 (0.82)	5.3 (5.0 to 6.0)	95	115	5.5 (0.87)	5.7 (5.0 to 6.0)
Environmental context and resources	1 to 7	97	129	6.5 (0.99)	7.0 (6.0 to 7.0)	95	115	6.4 (1.01)	7.0 (6.0 to 7.0)
Memory	1 to 7	97	129	5.7 (1.49)	6.0 (5.0 to 7.0)	95	115	5.6 (1.61)	6.0 (5.0 to 7.0)

sd = Standard deviation; IQR = Interquartile range

[a] For all outcomes (except fear avoidance beliefs) a larger score indicates greater agreement or likelihood in the clinicians' intentions and beliefs in performing the particular behaviour (i.e. not referring for plain X-ray or advising patients to stay active).

[b] The Knowledge variable was coded as indicating inadequate (0) or adequate (1) knowledge about key messages of the guideline.
